# Platform study of circulating tumor DNA directed adjuvant chemotherapy in colon cancer (CLAUDIA colon cancer, KCSG CO22-12)

**DOI:** 10.1186/s12885-025-14746-0

**Published:** 2025-08-25

**Authors:** Yongjun Cha, Sang-Hee Cho, Eun Young Park, Dong-Eun Lee, Taekeun Park, Moon Ki Choi, In Gyu Hwang, Seung-Hoon Beom, Byung Woog Kang, Jin-Soo Kim, Sun Young Kim, Seung Tae Kim, Seok Yun Kang, Jin Won Kim, Sae-Won Han, the Korean Cancer Study Group

**Affiliations:** 1 Division of Medical Oncology, Center for Colorectal Cancer, National Cancer Center, Goyang, Korea; 2 Department of Internal Medicine, Chonnam National University Hwasun Hospital, Hwasun, Korea; 3 Biostatistics Collaboration Team, Research Core Center, National Cancer Center, Goyang, Korea; 4 Department of Internal Medicine, Seoul National University Hospital, and Seoul National University Cancer Research Institute, Seoul, Korea; 5 Department of Internal Medicine, Chung-Ang University Hospital, Chung-Ang University College of Medicine, Seoul, Korea; 6 Department of Internal Medicine, Yonsei Cancer Center, Seoul, Korea; 7 Department of Oncology/Hematology, Kyungpook National University Hospital, Daegu, Korea; 8 Department of Internal Medicine, Seoul Metropolitan Government Seoul National University Boramae Medical Center, Seoul, Korea; 9 Department of Oncology, Asan Medical Center, Seoul, Korea; 10 Department of Medicine, Division of Hematology-Oncology, Samsung Medical Center, Sungkyunkwan University School of Medicine, Seoul, Korea; 11 Department of Hematology-Oncology, Ajou University School of Medicine, Suwon, Korea; 12 Department of Internal Medicine, Seoul National University Bundang Hospital, Seongnam, Korea

**Keywords:** Circulating tumor DNA, Colon cancer, Adjuvant chemotherapy, Minimal residual disease

## Abstract

**Background:**

Tumor-informed circulating tumor DNA (ctDNA) analysis allows for the sensitive detection of minimal residual disease (MRD) and has the potential to enhance patient stratification for adjuvant chemotherapy. We hypothesize that intensifying adjuvant chemotherapy in colon cancer patients with postoperative MRD positivity may reduce recurrence and improve survival outcomes.

**Methods:**

This multi-center platform trial (NCT05534087) consists of a prospective observational study (Part 1) and an interventional randomized trial (Part 2). In Part 1, approximately 1,200 patients with colon cancer will be screened for MRD at 3–6 weeks postoperatively using a tumor-informed, hybrid-capture-based ctDNA MRD assay that tracks up to 100 patient-specific somatic variants identified through tumor whole-exome sequencing.

Key eligibility criteria includes: age ≥ 19 years, ≤ 6 weeks post-curative resection, pathological diagnosis of colon adenocarcinoma, stage III or stage II with high-risk features requiring adjuvant chemotherapy with FOLFOX/CAPOX, and no macroscopic residual disease. All patients in Part 1 will complete 3 months of standard adjuvant FOLFOX/CAPOX while awaiting MRD results. Patients with MRD positivity will be screened for the Part 2 clinical trial following the completion of the initial 3 months of treatment titled “*Randomized Controlled Phase III Trial of Treatment Intensification in Stage II–III Colon Cancer Patients with Positive MRD after Curative Resection.*” MRD-negative patients are managed at the investigator’s discretion.

Part 2 investigates the superiority of an experimental arm (modified FOLFIRINOX for 3 months) compared to a control arm (FOLFOX/CAPOX for 3 months). The primary endpoint of the Part 2 randomized trial is the 3-year disease-free survival (DFS), while secondary endpoints include the 5-year overall survival, 5-year DFS, treatment-related adverse events, treatment compliance, and patient-reported outcomes. A total of 236 patients will be enrolled and randomized in a 1:1 ratio, assuming a hazard ratio of 0.64, 80% power, a two-sided alpha of 0.05, and a 10% dropout rate.

**Discussion:**

This trial will evaluate the effect of adjuvant chemotherapy intensification in colon cancer patients who are MRD-positive after curative surgery. This will enable a personalized adjuvant chemotherapy strategy based on postoperative MRD assessment in colon cancer.

**Trial registration:**

ClinicalTrials.gov: NCT05534087.

Clinical Research Information Service: KCT0007644.

## Background

The standard treatment for patients with high-risk stage II and stage III colon cancer consists of curative surgery followed by adjuvant chemotherapy with fluoropyrimidines and oxaliplatin [1, 2]. Although most patients are treated based on pathological stage and clinicopathological risk factors for recurrence, it is estimated that approximately half of patients with stage III colon cancer are cured by surgery alone, while only about one-quarter truly benefit from adjuvant chemotherapy [3, 4].

Recent studies across various solid tumors have demonstrated that circulating tumor DNA (ctDNA) analysis can detect minimal residual disease (MRD) even in the absence of radiographic or clinical evidence of disease, thereby substantially improving the prediction of recurrence [5–10]. Among patients with stage III colon cancer who receive standard adjuvant chemotherapy (FOLFOX or CAPOX) after curative resection, the 3-year disease-free survival (DFS) rate is approximately 75% [11, 12]. However, although research on ctDNA-guided MRD in colon cancer is still emerging, recent studies suggest that postoperative MRD-positive patients with stage II-III disease may have a 3-year DFS of around 30% even after standard adjuvant therapy and could account for more than half of all relapses among treated patient [5–7, 10]. Despite this high-risk profile, the current standard of care for MRD-positive patients remains 3–6 months of FOLFOX or CAPOX, regardless of MRD status [1, 2].

In this context, we designed a prospective, multi-center platform study (CLAUDIA Colon Cancer, KCSG CO22-12) to better characterize clinical outcomes in colon cancer patients according to their postoperative MRD status and to evaluate the clinical utility of MRD-informed treatment decisions in a controlled trial setting. The study comprises two parts: a prospective observational cohort (Part 1) and an interventional randomized clinical trial (Part 2). The platform design was adopted to allow for the future integration of additional MRD-guided interventional trials, reflecting the growing potential of MRD-based strategies in colorectal cancer management [13].

Currently, within Part 2, we are conducting a randomized controlled trial testing the hypothesis that incorporating a 3-month course of intensified treatment with modified FOLFIRINOX (mFOLFIRINOX) following an initial 3 months of standard FOLFOX or CAPOX may significantly improve 3-year DFS in MRD-positive patients with stage II–III colon cancer after curative resection.

## Methods

### Study aim and design

**Fig. 1:**
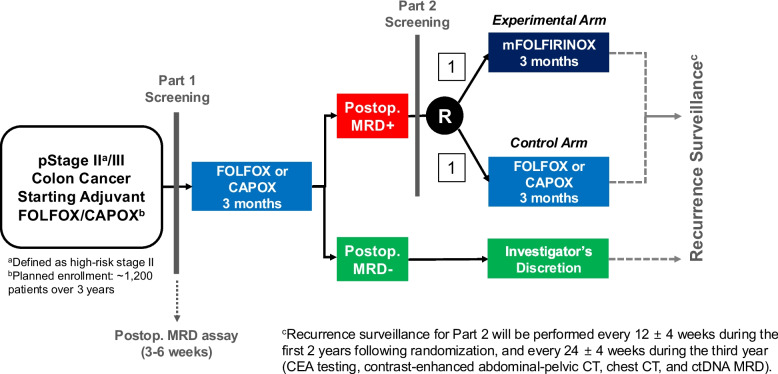
Overall study schema illustrating Part 1 and Part 2 of the study. Abbreviations: MRD, minimal residual disease; CEA, carcinoembryonic antigen; ctDNA, circulating tumor DNA

**Fig. 2:**
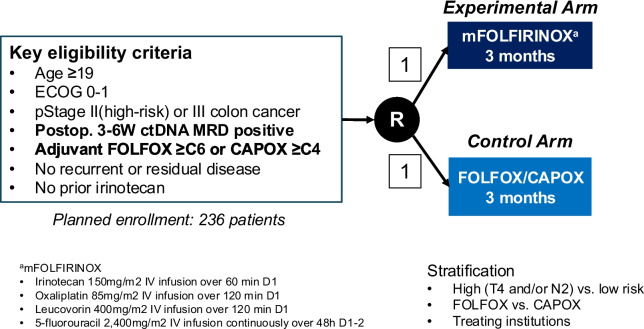
Detailed schematic of the Part 2 study design. Abbreviations: MRD, minimal residual disease; CEA, carcinoembryonic antigen; ctDNA, circulating tumor DNA

This multi-center, prospective, open-label, platform trial consists of two sequential parts: a prospective observational study (Part 1) and a randomized controlled phase III clinical trial (Part 2) (Fig. 1). Part 1 involves screening approximately 1,200 patients for MRD using postoperative ctDNA analysis, while Part 2 evaluates the efficacy of intensified chemotherapy (3 months of FOLFOX or CAPOX followed by 3 months of mFOLFIRINOX) versus standard chemotherapy (6 months of FOLFOX or CAPOX) in MRD-positive patients identified in Part 1 (Fig. 2).

### Participants

Eligible patients for Part 1 observational study are adults aged ≥ 19 years with histologically confirmed stage III or high-risk stage II colon adenocarcinoma who underwent curative surgical resection (R0) within the past 6 weeks and plan to receive standard adjuvant chemotherapy (FOLFOX or CAPOX). For stage II colon cancer, high-risk was defined as having ≥ 1 risk factor: pathological T4; poorly differentiated/undifferentiated histology; lymphatic/vascular invasion; perineural invasion; < 12 lymph nodes examined; bowel obstruction; localized perforation; close, indeterminate, positive margins; or tumor budding. Additional eligibility criteria include no macroscopic metastatic or residual disease on both abdomen-pelvis and chest CT taken within 4 weeks before or after surgery, adequate organ function, ECOG performance status 0–1 (0 for ≥ 70 years), and no prior chemotherapy or radiotherapy for colon cancer. Detailed eligibility criteria are listed in Table 1.

**Table 1:** Detailed patient eligibility criteria for part 1 and 2 studies

Inclusion criteria	Exclusion criteria
1. Written informed consent 2. Age ≥ 19 years 3. ECOG performance status 0–1 (ECOG 0 required if age ≥ 70) 4. Histologically confirmed adenocarcinoma of the colon (including rectosigmoid colon above the peritoneal reflection) 5. Pathological stage II–III colon cancer with high-risk features, including one or more of the following: • T4 lesion • Poor differentiation • Lymphatic or vascular invasion • Bowel obstruction • Fewer than 12 lymph nodes examined • Perineural invasion • Tumor perforation • Positive resection margins • Tumor budding 6. No evidence of distant metastasis on imaging (contrast-enhanced CT of the abdomen/pelvis and chest) performed within 4 weeks before or after surgery 7. apable and willing to comply with study procedures until study completion **Part 1–Specific Criteria:** 8. Planned initiation of adjuvant chemotherapy with FOLFOX or CAPOX within 6 weeks after curative (R0) resection 9. Agreement to participate in Part 2 if postoperative ctDNA testing indicates MRD positivity **Part 2–Specific Criteria:** 10. Completion of 6 cycles of FOLFOX or 4 cycles of CAPOX as adjuvant chemotherapy following R0 resection 11. Confirmed minimal residual disease (MRD) based on ctDNA testing performed 3–6 weeks postoperatively 12. Adequate bone marrow function: • ANC ≥ 1,300/µL • Platelets ≥ 75,000/µL • Hemoglobin ≥ 8.0 g/dL (patients requiring intermittent transfusions may still be eligible) 13. Adequate hepatic function: • Total bilirubin ≤ 1.5 × ULN • AST and ALT ≤ 3 × ULN 14. Adequate renal function: • Serum creatinine ≤ 1.5 × ULN • Creatinine clearance ≥ 50 mL/min	1. Refusal to participate in, or medical unsuitability for, the Part 2 trial (mFOLFIRINOX therapy) 2. Non-adenocarcinoma histology 3. Planned adjuvant chemotherapy that does not include oxaliplatin 4. Prior chemotherapy or radiotherapy before or after curative resection 5. History of other malignancies within the past 3 years, except for completely treated basal cell carcinoma of the skin, carcinoma in situ of the cervix, or thyroid cancer 6. Incomplete resection (R1 or R2 resection) 7. Evidence of recurrence or residual tumor on imaging or clinical examination 8. Diagnosis of familial adenomatous polyposis (FAP) or other polyposis syndromes 9. Diagnosis of two or more synchronous colon cancers of clinical or pathological stage II or higher, either concurrently or within the past 3 years 10. Pregnancy or lactation 11. Uncontrolled serious infections or other uncontrolled comorbidities 12. Significant or unstable pre-existing medical or psychiatric conditions that, in the opinion of the investigator, may compromise patient safety during the study **Part 2–Specific Criteria:** 13. Sexually active males and females of childbearing potential who are unwilling to use effective contraception during the treatment period and for 6 months after the last dose 14. Clinically significant cardiovascular disease, including: • Unstable angina requiring treatment • Symptomatic coronary artery disease • Congestive heart failure (NYHA class II or higher) • Serious cardiac arrhythmias • Acute coronary syndrome (e.g., myocardial infarction) within the past 6 months 15. Active viral infections such as HIV (Note: hepatitis B carriers may be eligible at the investigator’s discretion, with prophylactic antiviral therapy permitted) 16. Symptomatic inflammatory bowel disease 17. History of allogeneic transplantation requiring immunosuppressive therapy 18. Participation in another clinical trial involving investigational drugs or devices after curative resection for colon cancer 19. Presence of grade ≥ 3 peripheral neuropathy according to CTCAE v5.0 20. History of severe unexpected adverse reactions to fluoropyrimidines or platinum agents (Patients with anticipated but manageable reactions may be eligible at the investigator’s discretion) 21. Known Gilbert’s syndrome, dihydropyrimidine dehydrogenase (DPD) deficiency, or homozygosity for UGT1A1*28 alleles

### Procedures and interventions

In Part 1, patients undergo ctDNA MRD testing (CancerDetect™, IMBdx Inc.) using 20 mL whole-blood sample collected 3–6 weeks after surgery [10, 14–16]. Whole-exome sequencing (WES) of the surgical tumor tissue and matched white blood cells is first performed to identify patient-specific somatic mutations. Based on these findings, a personalized target capture panel comprising up to 100 variants is designed. ctDNA MRD testing is then conducted by high-depth sequencing of postoperative plasma DNA using this customized panel.

All enrolled patients initially receive 3 months of standard chemotherapy (FOLFOX/CAPOX). MRD test results will be disclosed to investigators and study participants within 3 months of initiating chemotherapy. For MRD-negative patients identified after completing the initial 3 months of standard chemotherapy, the decision to continue with an additional 3 months of chemotherapy (to complete a 6-month course) or to stop after 3 months will be left to the investigator’s discretion. MRD-positive patients will be screened for participation in Part 2 of the study after completion of the 3 months of chemotherapy. A total of 236 MRD-positive patients will be randomized 1:1 to receive either intensified chemotherapy (3 months of mFOLFIRINOX) or continued standard chemotherapy (an additional 3 months of FOLFOX or CAPOX).

The mFOLFIRINOX regimen consists of oxaliplatin (85 mg/m^2^), leucovorin (400 mg/m^2^), irinotecan (150 mg/m^2^), and 5-fluorouracil (2,400 mg/m^2^ for 46 h as a continuous infusion). The first dose of the clinical trial treatment is recommended to be administered on the scheduled start date of the next cycle of adjuvant chemotherapy—cycle 7 for FOLFOX or cycle 5 for CAPOX—and must be given within 3 weeks following randomization. Administration within + 7 days from Day 1 of each treatment cycle is permitted.

Stratified randomization was employed using mixed block sizes of 2 and 4 within each stratum. Patients are assigned through the web-based clinical trial platform system called Mytrial. The stratification factors were:

•Risk group: high-risk (stage III colon cancer with T4 and/or N2) vs. low-risk (stage III without T4/N2 or stage II colon cancer)
•Adjuvant chemotherapy regimen: FOLFOX vs. CAPOX
•Study site

**Fig. 3:**
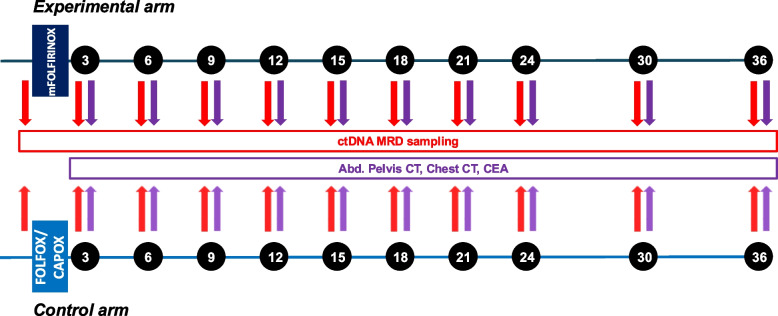
Recurrence surveillance protocol in the Part 2 study

For patients enrolled in Part 2, recurrence surveillance will be performed every 12 ± 4 weeks during the first 2 years following randomization, and every 24 ± 4 weeks during the third year. Surveillance will include carcinoembryonic antigen (CEA) testing, contrast-enhanced abdominal/pelvic CT, chest CT, and serial ctDNA MRD assessment using 20 mL whole-blood samples (Part 2, Fig. 3).

**Fig. 4:**
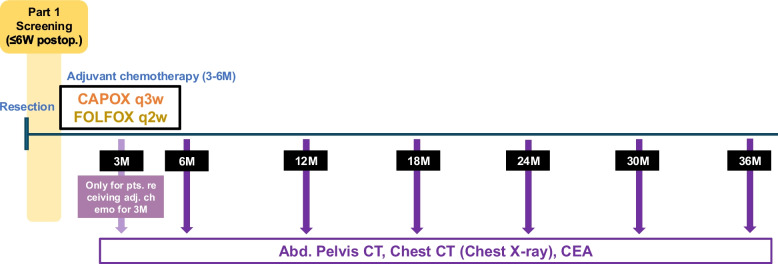
Recurrence surveillance protocol in the Part 1 study

MRD-negative patients, as well as MRD-positive patients who are ineligible for or decline participation in Part 2, will remain in the Part 1 observational study and undergo routine surveillance. In this group, recurrence monitoring will be conducted every 24 ± 4 weeks for a total of 3 years and will include CEA testing, contrast-enhanced abdominal/pelvic CT, and chest imaging with either CT or X-ray (Fig. 4).

For both group groups, if recurrence is confirmed during surveillance—or if a patient receives other treatments or participates in another clinical trial that may impact this study—recurrence surveillance will be discontinued.

After completion of the 3-year recurrence surveillance period, all patients will enter long-term follow-up and be monitored for survival every 6 months until death or the end of the study.

### Study endpoints

The primary endpoint of the Part 1 observational study is the proportion of patients who proceed to enrollment in the Part 2 interventional trial. Secondary endpoints include the rate of postoperative ctDNA MRD positivity, recurrence rates stratified by ctDNA MRD status, and recurrence rates according to digital pathology findings.

The primary endpoint of the Part 2 randomized trial is the 3-year DFS, defined as the time from the date of randomization to the date of first documented disease recurrence or death from any cause, whichever occurs first, with censoring at 3 years. The occurrence of second primary cancers will not be considered an event.

Secondary endpoints include 5-year overall survival (OS), defined as the time from the date of randomization to the date of death from any cause, with censoring at 5 years; 5-year DFS; treatment-related adverse events based on NCI-CTCAE v5.0; treatment compliance; and patient-reported outcomes, assessed using the EORTC QLQ-C30 questionnaire. Additionally, ctDNA dynamics—including clearance rate—will be assessed as exploratory endpoints.

### Statistical analysis

A total sample size of 236 patients (118 per arm) of the Part 2 study was calculated based on an assumed hazard ratio (HR) of 0.64 for 3-year DFS, providing 80% power with a two-sided alpha of 0.05, and accounting for a 10% dropout rate. This HR corresponds to an estimated 3-year DFS rate of 46.3% in the experimental arm and 30.0% in the control arm, reflecting a clinically meaningful and statistically significant absolute improvement of 16%.

Assuming uniform accrual over 3 years and a minimum follow-up of 3 years for the last enrolled patient, a total of 159 DFS events is required. One interim efficacy analysis is planned after 30% of events have occurred. Sample size estimation was performed using the rpact package in R (version 4.1.1).

Survival outcomes (DFS and OS) will be analyzed using the Kaplan–Meier method and compared between treatment groups using the stratified log-rank test, with stratification factors identical to those used at randomization. Treatment effects will be estimated using a stratified Cox proportional hazards model to calculate the HR and its corresponding two-sided 95% confidence interval (CI).

Efficacy analyses for Part 2 will be performed primarily using the Full Analysis Set (FAS), defined as a modified intention-to-treat (mITT) population that includes all randomized patients who have received at least one dose of the investigational product, in accordance with the ITT principle. Patients will be excluded from the FAS if they (1) do not meet key eligibility criteria, (2) have not received any investigational product, or (3) have not undergone at least one post-randomization assessment.

Sensitivity analyses will be conducted on the Per Protocol Set, a subset of the FAS that includes patients who adhere to the protocol. Safety analyses will be based on the Safety Set, defined as all patients who received at least one dose of the investigational product.

At the time of study design, large-scale data on postoperative ctDNA positivity rates and long-term survival outcomes by MRD status in colon cancer were limited. Accordingly, key design parameters, including the initial sample size, were based on short-term data. As external and internal evidence accumulates, an interim analysis is planned to (1) assess the validity of the assumptions used for the initial sample size calculation and (2) determine whether sample size re-estimation is necessary. The interim analysis will evaluate the primary endpoint (DFS) and safety, and specific procedures will be outlined in the Statistical Analysis Plan, version 1.2 (July 2025). This analysis will be conducted once at least 48 DFS events (30% of the planned 159) have occurred.

For all other analyses, statistical significance will generally be declared at a two-sided *p*-value < 0.050.

## Discussion

This study was registered at ClinicalTrials.gov (NCT05534087), with a registration date of September 9, 2022. This study has been ongoing since December 2022 across 11 Korean Cancer Study Group (KCSG) institutions. As of April 2025, 701 of the planned ~ 1,200 subjects have been enrolled in Part 1, and 116 of 236 subjects have been enrolled in Part 2.

In our study, we employed the modified FOLFIRINOX regimen as an intensification strategy. FOLFIRINOX is a combination chemotherapy regimen comprising three key agents—5-fluorouracil, oxaliplatin, and irinotecan—commonly used in the treatment of colorectal cancer. A similar regimen, FOLFOXIRI plus bevacizumab, demonstrated significantly improved response rates, progression-free survival, and OS compared to sequential doublet therapies (FOLFIRI or FOLFOX plus bevacizumab) in the phase III TRIBE and TRIBE2 trials [17, 18]. These results established FOLFOXIRI as a standard treatment option in metastatic colorectal cancer, particularly in patients with poor prognostic features [1, 19].

Beyond the metastatic setting, mFOLFIRINOX has shown clinical benefit in locally advanced rectal cancer. In the phase III UNICANCER-PRODIGE 23 trial, 3 months of neoadjuvant mFOLFIRINOX significantly improved pathological complete response rates, 3-year DFS, and 3-year metastasis-free survival compared to standard therapy [20]. Although triplet regimens were associated with increased rates of adverse events—such as diarrhea, mucositis, neutropenia, and febrile neutropenia—these toxicities were manageable and did not increase treatment-related mortality.

mFOLFIRINOX has also demonstrated efficacy in the adjuvant setting. In the PRODIGE 24/CCTG PA.6 trial, it significantly improved both DFS and OS compared to gemcitabine monotherapy in patients with resected pancreatic cancer [21], further supporting its use as an effective adjuvant regimen.

Based on this body of evidence, we consider mFOLFIRINOX a rational intensification strategy for patients with stage II–III colon cancer and molecular evidence of MRD following curative resection.

In our study, all patients initiate standard adjuvant FOLFOX or CAPOX within 3–6 weeks of surgery and continue for the first 3 months. While early initiation of intensified treatment based on MRD—as employed in trials such as NRG-GI008 (CIRCULATE-North America) [22, 23]—is a promising strategy, tumor-informed ctDNA assays commonly used to detect MRD require WES of tumor tissue, which typically takes 2–4 weeks. Consequently, waiting for MRD results prior to starting adjuvant therapy may delay treatment and increase the risk of recurrence [24].

To avoid this delay, our study adopts an alternative approach: all patients begin standard adjuvant chemotherapy without waiting for MRD results, and treatment is escalated in ctDNA-positive patients after the initial 3-month period (delayed intensification strategy). This approach ensures timely initiation of therapy while allowing for risk-adapted intensification in MRD-positive patients.

In our study, we chose to investigate an MRD-based intensification strategy for MRD-positive patients with stage II–III colon cancer, rather than a de-intensification approach for MRD-negative patients. This decision reflects our view that, given the current sensitivity limitations of MRD assays, intensification in MRD-positive patients is currently a more valuable area of investigation.

Several MRD-guided interventional trials are underway in patients with stage II–III colorectal cancer. The prospective observational GALAXY study, which evaluated MRD across stage I–IV colorectal cancer, demonstrated the strong prognostic significance of postoperative MRD detection (HR, 11.99; 95% CI, 10.02–14.35) [7, 25]. In this study, which utilized Natera’s Signatera™ assay, 19.2% (162/845) of patients with stage III colon cancer were MRD-positive at the postoperative timepoint, corresponding to a HR of 10.57 (95% CI, 7.94–14.07).

In our cohort, the postoperative MRD positivity rate was 28.3% (176/619) among patients with high-risk stage II and stage III colon cancer. Considering that approximately half of patients with stage III disease are expected to be cured by surgery alone without adjuvant chemotherapy, the theoretical maximum for MRD positivity could approach 50% if assay sensitivity were sufficient to detect all cases of residual disease [3, 4].

Although we have not yet reported the MRD positivity rate specifically for stage III patients, the overall positivity rate of 28.3% observed in our study—along with rates reported in other studies, including GALAXY—suggests that the current sensitivity of MRD assays may still be insufficient to justify safely omitting adjuvant chemotherapy in stage III patients based solely on postoperative MRD negativity.

In addition, the finding from GALAXY study that, among patients with high-risk stage II and III colon cancer, the clinical recurrence rate among ctDNA-negative patients was 9.63% in the adjuvant chemotherapy group and 8.53% in the observation group—and that 44% of all recurrences occurred in ctDNA-negative patients—suggests that current MRD assays alone may fail to identify a substantial proportion of patients at risk of recurrence [7, 25].

Based on these considerations, our study was designed to evaluate the benefit of treatment intensification in MRD-positive patients, rather than to test the safety of de-escalating therapy in MRD-negative patients with stage III colon cancer.

Although the recent ALTAIR trial—a pioneering MRD-based intensification study—showed no overall DFS benefit of trifluridine/tipiracil (FTD/TPI) versus placebo in MRD-positive colon cancer patients, a benefit was suggested in the stage IV subgroup [26]. However, this may reflect delayed recurrence rather than true MRD eradication, as the addition of a single-agent fluoropyrimidine to standard oxaliplatin-based adjuvant therapy may be insufficient. Additionally, a post-hoc ctDNA analysis from CALGB/SWOG 80702 suggested that celecoxib may provide DFS benefit specifically in MRD-positive patients, highlighting the potential of MRD as a predictive biomarker [27]. Therefore, we believe that MRD-based treatment intensification may lead to better outcomes.

In addition to our study, several ongoing escalation trials—such as NRG-GI008, AGITG DYNAMIC-III, CIRCULATE AIO-KRK-0217, CIRCULATE PRODIGE-70—are underway. The collective findings from these trials including ours will help inform the clinical utility of MRD-based escalation strategies in colon cancer [22, 23, 28–30].

However, for MRD-based adjuvant treatment strategies to become truly practice-changing for the broader patient population, MRD-guided de-escalation may be even more critical. In this regard, the recently reported post-hoc ctDNA analysis from the PRODIGE-GERCOR IDEA-France and HORG-IDEA-Greece trials is particularly noteworthy [24]. In their analysis, ctDNA positivity was observed in 19.7% (109/554) of patients with stage III colon cancer. While MRD positivity was strongly associated with worse DFS (HR, 5.75), clinicopathological high- and low-risk features (T4 or N2 vs. T1–3N1) remained independently prognostic regardless of MRD status. Furthermore, longer duration (six months vs. three months) of adjuvant oxaliplatin-based chemotherapy continued to demonstrate improved DFS in both MRD-positive and MRD-negative groups. The impact of chemotherapy duration appeared to diminish only among ctDNA-negative patients with low-risk (T1–3/N1) disease. These findings suggest that, in the absence of highly sensitive MRD assays, it may still be reasonable to integrate MRD status with conventional clinicopathological risk factors for treatment decision-making, particularly when considering de-escalation.

Recent efforts, such as whole-genome sequencing–based discovery of tumor-specific alterations and the tracking of up to ~ 1,800 mutations using personalized panels in patients with lung adenocarcinoma and breast cancer, have suggested that this approach may enhance assay sensitivity [8, 9]. Whether our understanding proves correct or not, we believe that improving the sensitivity of MRD assays is the most critical factor for their broader adoption in clinical practice, particularly for MRD-guided de-escalation strategies. Several ongoing clinical trials, including NRG-GI008 and VEGA, are currently evaluating the clinical utility of de-escalation protocols in postoperative MRD-negative patients, and their results are eagerly awaited [22, 23, 25].

The clinical implications of MRD detection are expected to be profound in the treatment of cancer patients. Our study is one of the earliest and largest clinical trials investigating the impact of MRD-guided treatment decisions in patients with resectable colon cancer and represents a significant collaborative effort within the oncology community. We believe that such efforts will contribute to refining patient stratification, enabling tailored therapeutic interventions that minimize unnecessary treatment toxicity while improving disease control and overall patient outcomes.

## Data Availability

No datasets were generated or analysed during the current study.

## Abbreviations

CAPOX: Capecitabine and oxaliplatin
CEA: Carcinoembryonic antigen
CI: Confidence interval
ctDNA: Circulating tumor DNA
DFS: Disease-free survival
ECOG: Eastern Cooperative Oncology Group
EORTC QLQ-C30: European Organization for Research and Treatment of Cancer Quality of Life Questionnaire-C30
FAS: Full Analysis Set
FOLFOX: 5-fluorouracil, leucovorin, and oxaliplatin
FOLFIRI: 5-fluorouracil, leucovorin, and irinotecan
FOLFOXIRI: 5-fluorouracil, leucovorin, oxaliplatin, and irinotecan
FTD/TPI: Trifluridine/tipiracil
HR: Hazard ratio
ITT: Intention-to-treat
KCSG: Korean Cancer Study Group
mFOLFIRINOX: Modified leucovorin, 5-fluorouracil, irinotecan, and oxaliplatin
MRD: Minimal residual disease
NCI-CTCAE: National Cancer Institute Common Terminology Criteria for Adverse Events
OS: Overall survival
WES: Whole-Exome Sequencing
